# Effect of health insurance type on health care utilization in patients with hypertension: a national health insurance database study in Korea

**DOI:** 10.1186/s12913-014-0570-9

**Published:** 2014-11-21

**Authors:** Hae Sun Suh, Hye-Young Kang, Jinkyung Kim, Euichul Shin

**Affiliations:** College of Pharmacy, Pusan National University, Busandaehak-ro 63 Beon-gil 2, Geumjeong-gu, Pusan, South Korea; College of Pharmacy, Yonsei Institute of Pharmaceutical Sciences, Yonsei University, 162-1 Songdo-Dong, Yeonsu-Gu, Incheon, 406-840 South Korea; Department of Hospital Administration, Konyang University, Gwanjeodong-ro 158, Seo-Gu, Daejeon 302-832 South Korea; Department of Preventive Medicine, College of Medicine, the Catholic University of Korea, Banpodae-ro 222, Seocho-Gu, Seoul 137-701 South Korea

**Keywords:** Hypertension, Insurance, Health, Costs and cost analysis, Hospitalization

## Abstract

**Background:**

Higher utilization of healthcare services has been observed among individuals who receive public aid compared to individuals who do not receive public aid in many countries. However, no systematic investigations have explored whether this pattern of higher utilization persists after correcting for a number of factors in Korea. In this study, we sought to examine whether the type of health insurance, wage-based contributory insurance (Health Insurance, HI) or government-subsidized public assistance (Medical Aid, MA), affects the utilization of inpatient services after controlling for baseline patient and institutional characteristics among patients with hypertension in Korea.

**Methods:**

The Korean National Health Insurance claims database from 2006 and 2007 was used for analysis. To avoid biased estimates, we determined the most appropriate type of multivariate model for each outcome variable: a logistic regression model for the likelihood of hospitalization, a zero-inflated negative binomial model for the length of stay (LOS), and a generalized linear model with a log-link function for hospitalization costs.

**Results:**

Adjusted odds ratio (OR) and factor changes showed that MA patients (n = 21,539) had a significantly higher likelihood of hospitalization (OR: 1.41-1.71), average LOS per patient (factor change: 1.31-1.42), and hospitalization costs per patient (factor change: 1.10-1.41) compared to HI patients (n = 304,027).

**Conclusions:**

The pattern of higher healthcare utilization among MA patients persists even after controlling for baseline health conditions. This finding confirms that the type of health insurance affects the utilization of healthcare resources, and suggests that effective strategies are necessary to prevent the potential overutilization of inpatient care by MA patients with hypertension in Korea.

## Background

One prominent adverse effect of providing health insurance, especially under fee-for-service payment systems, is the overutilization of care [1]. Beneficiaries of health insurance without or with low co-payments are likely to use more healthcare resources compared to those paying higher co-payments [2,3]. This phenomenon has been observed in Korea [4,5].

Korea has two tiers of the universal health insurance system: the Health Insurance (HI) and the Medical Aid (MA) program. The HI program is a wage-based, contributory insurance program covering about 96% of the population, and the MA program is a government-subsidized public assistance program for poor and medically indigent individuals [6]. Unlike many countries such as the United States, the United Kingdom, Spain, Italy, and Denmark, patients in Korea can consult specialists directly without seeing a gatekeeper [7]. With no strict gate-keeping system, patients can easily access secondary care in tertiary hospitals, and there are no differences in the benefits provided to beneficiaries in the HI and the MA programs [8].

According to the 2010 National Health Insurance statistics, the per-capita utilization and cost of insurance-covered services have been consistently higher among those enrolled in the MA program compared to those in the HI program. The average medical expenses per beneficiary are three times higher and the average length of stay is twice longer in the MA group than in the HI group [4]. This raises a concern that individuals with MA insurance might over-utilize healthcare and incur unnecessary costs due to low co-payments or waivers of co-payment. Conversely, some argue that those enrolled in public assistance health insurance programs such as the MA program use healthcare services more because people with a low socioeconomic status tend to have poorer health and are more susceptible to illness [9]. However, there have been no systematic investigations as to whether the higher utilization by individuals with MA insurance in Korea is due to poorer health or greater use of healthcare resources.

Therefore, we compared the hospitalization rates related to hypertension between the HI and the MA groups in Korea in an attempt to assess whether beneficiaries of the MA program have higher utilization rates of inpatient care than those in the HI program after controlling for baseline health conditions. Hypertension is one of the ‘ambulatory care sensitive conditions (ACSC)’, which have been defined as health conditions for which hospitalization can be avoided through timely and effective primary care in outpatient settings [10,11]. Per the definition of ACSC, it is not common for patients with hypertension to be hospitalized, as the blood pressure is usually managed through medication therapy in an outpatient setting. Thus, we selected hypertension in order to study the possibility of unnecessary use of inpatient services. Besides, hypertension was selected for this comparison because it is a major risk factor for cardiovascular disease, which is the second highest cause of mortality in Korea [12]. The disease burden of hypertension is expected to increase rapidly due to the aging of the Korean population, and the amount of treatment for hypertension ranks among the highest [4,5,13,14]. Therefore, a critical need exists to develop effective treatment strategies to control hypertension and to efficiently allocate healthcare resources. We hypothesized that if higher admission rates by MA patients persisted after adjustment for differences in baseline health conditions and characteristics between the two groups, this would indicate a potential overutilization of inpatient care for hypertension among the MA group.

## Methods

### Data sources and study subjects

**Figure 1:**
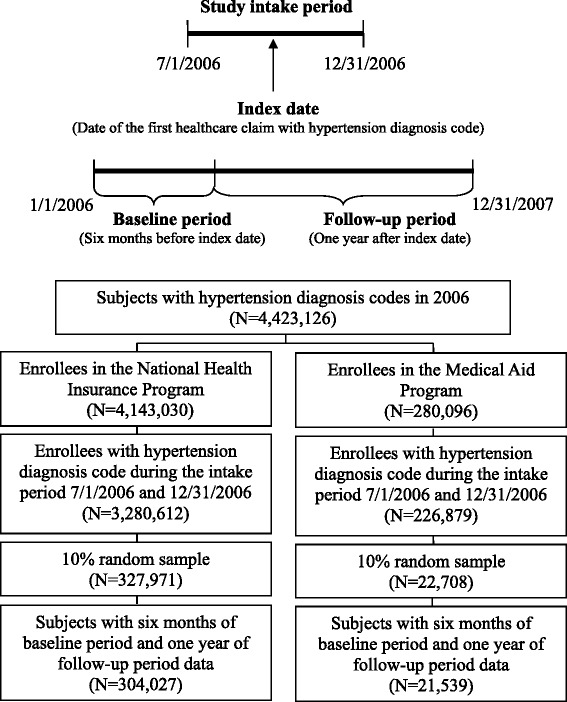
**Study time period and identification of the target population.**

We have used the Korean National Health Insurance (NHI) claims database obtained through a formal request to the Health Insurance Review and Assessment Service in Korea for research purpose because the database was not publicly available [15]. Records from the Korean NHI claims database for two years, 2006 and 2007, were used to identify HI and MA patients with hypertension and to compare their healthcare utilization. We used all claims records for enrollees of the HI and the MA programs who were continuously enrolled between 2006 and 2007 and were aged 19 or older at the index date. Patients were identified as having hypertension if they had at least one claim record (i.e., inpatient, outpatient, or emergency department visit) with a primary, secondary, tertiary, or quaternary diagnosis of hypertension (*International Classification of Diseases, Tenth Revision, Clinical Modification* (ICD-10-CM) billing codes of I10.x, I11.9, and I13.9) during a six-month intake period from July 1 to December 31, 2006. Figure 1 depicts the three different observation periods employed in this study: baseline, intake, and follow-up period. The date of the first claim of hypertension during the intake period was defined as the index date for each patient. For the efficiency of the data analysis, we randomly sampled 10% of the identified patients in the HI and the MA group.

Figure 1 presents the sampling process used to identify the target population. For each of the sampled patients, baseline characteristics such as overall healthcare utilization and comorbid conditions were observed for a six-month baseline period before the index date to adjust for baseline disease severity. In addition, hypertension-related healthcare utilization, defined as any outpatient, inpatient, or emergency department visit with a primary, secondary, tertiary, or quaternary diagnosis of hypertension was tracked for each patient for a one-year follow-up period after his or her index date. As a result, a total of 325,566 patients were included in the data analysis, composed of patients enrolled in the HI program (n =304,027) and in the MA program (n =21,539).

### Study variables

#### Dependent variables

We defined three outcome measures as dependent variables to compare the likelihood and the extent of utilization of inpatient services between HI and MA patients with hypertension: hospital admission, length of stay (LOS), and hospitalization costs related to hypertension. The likelihood of being hospitalized with hypertension was operationalized as any occurrence of hospital admission with a primary, secondary, tertiary, or quaternary diagnosis of hypertension during the one-year follow-up period. The extent of inpatient services utilized was measured as the total LOS and the total insurance-covered medical costs of hospital admissions for hypertension per patient during the one-year follow-up period. For patients without any hospital admission for hypertension, these two variables were given zero values. The insurance-covered medical costs included both the insurer’s payment and patient’s co-payment for the services listed on the NHI-covered services, and captured all types of medical services as long as they were covered by the NHI, such as charges for diagnostic tests, medical procedures and pharmaceuticals, and the accommodation costs for rooms and meals during hospitalization.

#### Independent variables

The major independent variable in this study is the health insurance type, HI or MA. The beneficiaries in the MA group were further categorized into two subgroups: Class I with almost no co-payment (due to not being able to work at all) and Class II with relatively higher co-payments than Class I (due to being able to work). We did not categorized the beneficiaries in the HI group as the MA group because the co-payment rate in beneficiaries with Health Insurance varies by types of services they used, types of institutions they visited, indications of services, etc.

We included selected baseline patients and institutional characteristics observed during the six-month baseline period as covariates. These characteristics are considered to influence the utilization of inpatient services related with hypertension. Patient characteristics included demographic characteristics at the index date (i.e., age and gender), overall health status measured by the Elixhauser comorbidity index, and total medical costs and total number of outpatient visits for all causes of hypertension during the baseline period. The Elixhauser index is a standard measure of comorbidity, which is a count variable indicating the number of medical conditions among 30 pre-defined comorbidities [16]. Index scores range from a minimum of 0 to a maximum possible value of 30, with higher comorbidity indices indicating sicker patients.

The characteristics of healthcare institutions at the index date are presented in terms of type of institution, ownership status, number of hospital beds, and location. According to Korean medical law, healthcare institutions are categorized into six types: clinic, public health center, hospital, general hospital, tertiary-care hospital, and long-term care facility. Ownership was classified into two types: government-owned and non-government owned hospitals. Location was categorized as the capital city of Seoul, six major cities, and nine provinces. Lastly, the healthcare institutions were categorized into five groups according to the number of hospital beds: <100, 100–299, 300–799, 800–999, and ≥1,000.

### Data analysis

We used descriptive statistics to compare the baseline characteristics between the HI and the MA groups. Two-tailed *t*-tests and *chi*-square tests were used to compare differences in means and proportions, respectively. We conducted all analyses using SAS statistical software version 9.2 (SAS Institute Inc., Cary, NC, USA) and STATA software version 10 (StataCorp LP, College Station, TX, USA).

#### Hospital admission

We developed a multivariate logistic regression model to assess whether there were significant differences in the likelihood of hypertension-associated hospital admission between HI and MA patients with hypertension after controlling for baseline patient and institutional characteristics. In addition to the Elixhauser comorbidity index used as a continuous variable, measures of overall healthcare utilization, such as the total insurance-covered medical costs and the total number of outpatient visits for all causes during the 6-month baseline period, were included in the model to adjust for patient baseline health status. The *c*-statistic was examined to assess the model performance [17]. We examined the variance inflation factors to check whether the model suffered from multicollinearity. A value above 10 was considered to indicate a serious multicollinearity problem [18].

#### Length-of-stay (LOS)

We used count data models such as Poisson and negative binomial regression models to compare the LOS between the HI and the MA group. Count data models are more appropriate than standard linear regression models for this outcome variable, which is a non-negative integer dependent variable [19]. The Poisson model implies the equality of the conditional variance and the conditional mean of the count variable, a property called equidispersion [20]. When the variance exceeds the mean, this overdispersion feature leads to inconsistent and inefficient estimates. The negative binomial model accounts for overdispersion by including the mean of the count variable as a random variable following a gamma distribution. Therefore, we employed the negative binomial model for the LOS variable because the unconditional variances of this variable were larger than the unconditional means. We also performed a likelihood ratio test of the overdispersion parameter alpha, where a significant p-value rejects the Poisson model in favor of the negative binomial model. In addition, because the distribution of the LOS was markedly skewed and the number of zeroes was excessive (93%), we employed the zero-inflated negative binomial model which accounts for both overdispersion and excess zeroes. We confirmed the use of the zero-inflated count model by performing the Vuong test [19,20]. A significant test result indicates that the zero-inflated count model has a better fit than the standard count model.

#### Costs

We developed a cost model to assess whether there were significant differences in the hospitalization costs between the HI and the MA group after controlling for covariates. Healthcare expenditure data is typically characterized by a nontrivial fraction of zero outcomes, highly skewed to the right of non-zero outcomes, and non-negative measurements of the outcomes [21]. If the ordinary least squares (OLS) method is used to examine the relationship between the type of insurance and hospitalization costs, this approach may yield biased estimates since the OLS method is very sensitive to outliers. In addition, erroneous predictions such as negative values may occur. Logarithmic transformation of expenditures can be used to mitigate the skewness problem, excluding observations with zero expenditure. However, this approach may also yield biased estimates, and therefore Duan’s smearing estimator is used when heteroscedasticity exists [22].

Because the distribution of the hospitalization cost variable was markedly skewed and heteroscedasticity existed in our cost data as assessed by the generalized linear model version of the Park test on the log-scale of hospitalization costs, we did not use the OLS method with logarithmic-transformed cost data, as otherwise the estimation results would have been biased [21]. Instead, we used an exponential conditional model (ECM) using a generalized linear model framework, where the central structure of the model is an exponential conditional mean, to estimate the factors influencing hospitalization costs, and control for baseline demographic, comorbidity, and hospital characteristics. The Box-Cox test and the modified Park test were used to find the appropriate link function and the distribution family for the hospitalization cost variable. We used the Pregibon link test to assess the model fit.

## Results

We have included patients with at least one claim record with hypertension. The minimum and maximum number of claims in HI group (N = 304,027) were 1 (274 cases, 9%) and 321 (1 case, 0.0003%), respectively. The minimum and maximum of claims in MA group (N = 21,539) were 1 (18 cases, 0.8357%) and 132 (1 case, 0.0046%), respectively. The mean number of claims in HI group and MA group were 16.1 (median 16.0) and 17.5 (median 17.0), respectively. The numbers of patients who were hospitalized were 18,937 (6.23%) and 3,677 (17.07%) in HI group and MA group, respectively.

Overall, enrollees in the two insurance programs appeared to be heterogeneous in terms of demographic characteristics and baseline health status (Table 1). Patients in the MA group were older and sicker compared to those in the HI group (p <0.0001). The average Elixhauser comorbidity index of the MA patients (3.0) was significantly higher than that of the HI patients (2.3), with a higher score indicating poorer health. The average insurance-covered medical costs for all causes and for hypertension-associated conditions among MA patients was about 2.37 and 2.19 times higher than among HI patients, respectively. More frequent ambulatory care visits for hypertension-associated conditions (1.07 times, 4.6 visits for MA and 4.3 visits for HI patients) as well as for all causes (1.13 times, 5.4 visits for MA and 4.8 visits for HI patients) were observed among beneficiaries of the MA program during the six-month baseline period.

**Table 1:** **Baseline characteristics of enrollees in the National Health Insurance and Medical Aid programs**

	**National Health Insurance (N = 304,027)**	**Medical Aid (N = 21,539)**	***p*** **-value**
***Patient characteristics during the 6-month baseline period***			
Medical Aid			
Class I^a^, n (%)	-	17,248 (80.1)	-
Class II^b^, n (%)	-	4,291 (19.9)	-
Age in years, mean (SD)	61.6 (11.7)	68.1 (11.9)	<0.0001
Male, n (%)	130,398 (42.9)	6,000 (27.9)	<0.0001
Insurance-covered medical costs in Korean won^c^, mean (SD)			
All causes	228,566 (838,665)	541,699 (1,359,656)	<0.0001
Hypertension related^d^	171,249 (616,724)	375,440 (995,523)	<0.0001
No. outpatient visits, mean (SD)			
All causes	4.8 (2.6)	5.4 (2.7)	<0.0001
Hypertension related	4.3 (2.3)	4.6 (2.0)	<0.0001
Elixhauser comorbidity index^e^, mean (SD)	2.3 (1.3)	3.0 (1.7)	<0.0001
n (%)			
1	103,722 (34.1)	4,074 (18.9)	<0.0001
2	92,830 (30.5)	5,614 (26.1)	
3	57,212 (18.8)	4,684 (21.8)	
4	28,815 (9.5)	3,160 (14.7)	
≥5	21,448 (7.1)	4,007 (18.6)	
***Characteristics of healthcare institution at index visit***			
Type of institution, n (%)			<0.0001
Tertiary-care hospital	19,101 (6.3)	536 (2.5)	
General hospital	28,703 (9.4)	3,586 (16.7)	
Hospital	14,174 (4.7)	2,185 (16.7)	
Long-term care facility	1,026 (0.3)	368 (1.7)	
Clinic	203,556 (67.0)	11,432 (53.1)	
Public health center	36,740 (12.1)	2,288 (10.6)	
Type of ownership, n (%)			<0.0001
Government owned	40,031 (13.2)	3,086 (14.3)	
Non-government owned	263,947 (86.8)	18,449 (85.7)	
Missing	49 (0.0)	4 (0.0)	
Bed size, n (%)			<0.0001
<100	248,378 (81.7)	15,818 (73.4)	
100 ~ 299	17,077 (5.6)	2,790 (13.0)	
300 ~ 799	24,925 (8.2)	2,281 (10.6)	
800 ~ 999	7,377 (2.4)	435 (2.0)	
≥1,000	6,270 (2.1)	215 (1.0)	
Location, n (%)			<0.0001
Seoul	72,002 (23.7)	3,190 (14.8)	
Six major cities	73,653 (24.2)	5,329 (24.7)	
Kwangju	7,457 (2.5)	761 (3.5)	
Daegu	14,106 (4.6)	1,056 (4.9)	
Daejeon	8,787 (2.9)	641 (3.0)	
Pusan	22,703 (7.5)	1,706 (7.9)	
Ulsan	5,261 (1.7)	264 (1.2)	
Incheon	15,339 (5.1)	901 (4.2)	
Nine provinces	158,323 (52.1)	13,016 (60.4)	
Kangwon	11,442 (3.8)	1,084 (5.0)	
Kyonggi	59,167 (19.5)	3,065 (14.2)	
Kyungsangnam	17,754 (5.8)	1,432 (6.7)	
Kyungsangbuk	16,935 (5.6)	1,680 (7.8)	
Jeollanam	13,100 (4.3)	1,921 (8.9)	
Jeollabuk	12,466 (4.1)	1,706 (7.9)	
Jeju	3,279 (1.1)	236 (1.1)	
Chungcheongnam	14,200 (4.7)	1,058 (4.9)	
Chungcheongbuk	9,980 (3.3)	834 (3.9)	
Missing	49 (0.0)	4 (0.0)	

^a^Beneficiaries of Medical Aid who are not able to work at all; ^b^Beneficiaries of Medical Aid who are able to work; ^c^All costs are presented in 2006 Korean currency value (1,200 Korean won [KRW] =1 US dollar); ^d^Hypertension-related visits are defined as visits with a primary, secondary, tertiary, or quaternary diagnosis of hypertension; ^e^The higher the comorbidity indices, the sicker patients are; SD = standard deviation.

The utilization of inpatient services related to hypertension during the one-year follow-up period was compared between HI and MA patients (Table 2). The MA group had a higher probability of hospitalization associated with hypertension compared to the HI group. About 170.71 patients per 1,000 hypertension patients enrolled in the MA program had at least one hypertension-associated hospitalization, a rate three times higher than for those enrolled in the HI program (62.29 per 1,000 hypertension patients). The average LOS per patient was also significantly higher in the MA group (6.04 days) compared with the HI group (1.27 days). On average, a patient enrolled in the MA program had hospitalization costs (507,192 Korean won (KRW), 1 US dollar =1,200 KRW) nearly three times higher than those in the HI program (175,892 KRW). Within the MA group, patients from the Class I group showed consistently higher utilization of inpatient services compared to those from the Class II group in terms of hospitalization rate, LOS, and costs.

**Table 2:** **Comparison of utilization of hypertension-related inpatient services during a one-year follow-up period between National Health Insurance and Medical Aid enrollees**

	**National Health Insurance (HI)**	**Medical Aid (MA)**		
		**All**	**Class I**	**Class II**
No. enrollees	304,027	21,539	17,248	4,291
No. patients hospitalized with HTN per 1,000 patients	62.29	170.71	186.69	106.50
Average LOS per patient in days (SD)	1.27	6.04	6.74	3.24
	(11.22)	(28.55)	(30.03)	(21.42)
Average cost for ICS per patient (SD)	175,892	507,192	562,100	286,482
	(1,136,418)	(1,921,397)	(2,008,176)	(1,503,610)
Average LOS per admission in days (SD)	11.15	12.83	12.98	11.79
	(11.19)	(10.94)	(10.99)	(10.49)
Average cost for ICS per admission (SD)	1,958,934	1,429,097	1,433,674	1,396,845
	(2,298,445)	(1,749,599)	(1,730,079)	(1,883,112)
Average LOS per patient hospitalized with HTN in days (SD)	20.39	35.39	36.09	30.42
	(40.38)	(61.14)	(61.40)	(59.07)
Average cost for ICS per patient hospitalized with HTN (SD)	2,823,890	2,971,010	3,010,903	2,689,922
	(3,640,973)	(3,782,638)	(3,772,490)	(3,845,845)

SD = standard deviation; LOS = length of stay; HTN = hypertension; ICS = insurance-covered services; All inpatient services were hypertension-related, with a primary, secondary, tertiary, or quaternary diagnosis of hypertension; All costs are presented in 2006 Korean currency value (1,200 Korean won [KRW] =1 US dollar).

The unit intensity of inpatient care measured as the average LOS and cost per admission was not consistently higher in the MA group compared to the HI group. MA patients stayed 15% longer (12.83 vs. 11.25 days), but spent 27% less during hospitalization (1,429,097 vs. 1,958,934 KRW).

For patients who had been hospitalized with hypertension at least once during the follow-up period, we conducted a subgroup analysis to examine the extent of inpatient service utilization among those experiencing hospitalization. For the MA group, the mean of the total LOS per patient for one year was 35.39 days, which leads to a calculation of 2.76 (=35.39/12.83) admissions per patient. For the HI group, the mean of the total LOS per patient was 20.39 days, indicating 1.83 (=20.39/11.25) admissions per patient. For the hospitalization cost variable, the difference between the two groups was very marginal, with a difference of only 1.05 times (2,823,890 KRW for the HI group and 2,971,010 KRW for the MA group).

We conducted a multivariate logistic regression analysis to examine whether the difference in the probability of hospital admission for hypertension between the two insurance groups remained after controlling for baseline characteristics (Table 3). Patients in the MA group had a greater likelihood of being hospitalized compared with patients in the HI group, as reflected in the significant odds ratio (OR) of 1.71 (95% CI =1.63-1.79) and 1.41 (95% CI =1.27-1.57) for the MA Class I and Class II groups, respectively. Older age, higher medical costs (all causes), fewer outpatient visits (all causes), and higher comorbidity were all significantly associated with a greater likelihood of hospital admission (p <0.0001). The c-statistic was 0.78, indicating that the model performed very well. The model did not suffer from a multicollinearity problem because all variance inflation factors were below 10 and tolerance values were above 0.1.

**Table 3:** **Regression analysis results for the association between type of health insurance and utilization of hypertension-related inpatient services among patients with hypertension**

	**Hospital admission**	**Length of stay**	**Hospitalization costs**
	**Odds ratio [95% CI]**	**Factor change** ^**a**^ **[95% CI]**	**Factor change** ^**a**^ **[95% CI]**
**Type of insurance**			
Medical Aid Class I^b^	1.710^***^	1.305^***^	1.412^***^
	[1.632-1.792]	[1.243-1.370]	[1.289-1.545]
Medical Aid Class II^c^	1.413^***^	1.419^***^	1.103
	[1.271-1.570]	[1.263-1.594]	[0.907-1.340]
Health Insurance [ref]	-	-	-
**Age**	1.029^***^	1.014^***^	1.037^***^
	[1.028-1.030]	[1.013-1.016]	[1.035-1.040]
**Gender**			
Male	0.999	0.992	0.893^**^
	[0.969-1.030]	[0.959-1.027]	[0.839-0.951]
Female [ref]	-	-	-
**Total costs for insurance-covered services (all causes)** ^**d**^	1.013^***^	1.012^***^	1.024^***^
	[1.012-1.014]	[1.011-1.013]	[1.022-1.027]
**Total No. outpatient visits (all causes)**	0.981^***^	0.970^***^	0.988^*^
	[0.975-0.986]	[0.966-0.974]	[0.978-0.998]
**Elixhauser comorbidity index**	1.577^***^	1.074^***^	1.784^***^
	[1.563-1.592]	[1.064-1.084]	[1.749-1.822]
**Ownership of institution**			
Non-government owned	1.004	0.891	1.197^*^
	[0.910-1.107]	[0.805-0.986]	[1.013-1.416]
Government owned [ref]	-	-	-
**Type of institution**			
Tertiary hospital	2.117^***^	0.544^***^	1.240^**^
	[2.005-2.235]	[0.505-0.585]	[1.091-1.409]
General hospital	1.923^***^	0.713^***^	1.065
	[1.844-2.006]	[0.670-0.758]	[0.949-1.194]
Hospital^e^ [ref]	2.240^***^	-	-
	[2.126-2.360]		
Long-term care facility	5.213^***^	2.919^***^	2. 474^***^
	[4.580-5.933]	[2.583-3.300]	[2.024-3.022]
Public health center	1.260^***^	0.696^***^	0.880
	[1.130-1.406]	[0.616-0.786]	[0.715-1.084]
Clinic^f^ [ref]	-	0.673^***^	0.549^***^
		[0.636-0.712]	[0.496-0.607]
**Location of institution**			
Six major cities	1.134^***^	1.358^***^	0.992
	[1.085-1.185]	[1.293-1.426]	[0.907-1.085]
Nine provinces	1.248^***^	1.145^***^	1.046
	[1.200-1.298]	[1.096-1.196]	[0.968-1.131]
Seoul [ref]	-	-	-
***c*** **-statistic**	0.778		
**Dispersion parameter**		1.481	
***P*** **-value for LR test** ^**g**^		< 0.0001	
**Vuongstatistic** ^**h**^		93.73^***^	

All inpatient services were hypertension-related, with a primary, secondary, tertiary, or quaternary diagnosis of hypertension; Robust standard errors in parenthesis; All covariates at index-date otherwise specified; ^a^Exp (coefficient estimate) = factor change in expected hospitalization costs for a unit increase in a covariate; ^b^Beneficiaries of Medical Aid who are not able to work at all; ^c^Beneficiaries of Medical Aid who are able to work; ^d^Insurance-covered medical costs during the 6-month baseline period in 2006 Korean won (one US dollar approximately equals 1,200 Korean won); ^e^Hospital used as a reference for length-of-stay and hospitalization costs variable; ^f^Clinic used as a reference for the hospital admission variable; ^g^Likelihood Ratio (LR) test comparing negative binomial model against Poisson model; ^h^Comparing zero-inflated negative binomial model against negative binomial model; * p <0.05; ** p <0.001; *** p <0.0001; SE = standard error; 95% CI =95% confidence interval.

To compare the extent of hypertension-associated inpatient services used between the two groups after adjusting for baseline characteristics, we employed a zero-inflated negative binomial count model for the total LOS per patient (Table 3). The estimated value of the overdispersion parameter alpha was positive (value =1.48; 95% CI =1.44-1.53) and the likelihood ratio test was significant, indicating that the negative binomial model was preferred to the Poisson model (p <0.0001). We had significant results for the Vuong test, which supported the zero-inflated negative binomial model over the negative binomial model for the LOS outcome (p <0.0001).

Among patients who were hospitalized when sick, the expected total LOS per patient in the MA Class I and Class II groups were 1.31 times (95% CI =1.24-1.37) and 1.42 times (95% CI =1.26-1.59) longer than in the HI group, respectively. Patients in the MA Class I and Class II groups had a significantly higher probability of being in the certain admission group compared with those in the HI group.

For the cost model examining the relationship between type of insurance and hospitalization costs, the Box-Cox test and the modified Park test indicated that the appropriate generalized linear model was a gamma distribution with a log link function. The Pregibon Link test showed that the model performed well (p =0.559). After controlling for covariates, the predicted incurred costs of hospitalization for the MA Class I group were approximately 41% higher than the costs for the HI group. However, the predicted costs for the MA Class II group were not significantly higher than those for the HI group.

## Discussion

In this study, we examined whether the type of health insurance, i.e. wage-based contributory insurance versus government-subsidized public assistance program, affects the extent of healthcare utilization under the universal health insurance system in Korea. While the beneficiaries of the contributory program (National Health Insurance) pay wage-based insurance premiums and out-of-pocket co-payments for healthcare services, the beneficiaries of the public assistance program (Medical Aid) do not pay insurance premiums and most of their co-payments are waived. The low prices or expenses paid by MA enrollees may lead to low concern for the efficient use of healthcare resources, resulting in higher utilization of healthcare services.

Our study results provide empirical evidence that hypertension patients in the MA program tend to utilize more healthcare resources for their inpatient care than those in the HI program. Patients in the MA program tended to get hospitalized more often, and incurred higher hospitalization costs and longer inpatient stays to treat hypertension-associated conditions than their counterparts from the HI program. This tendency persisted even after controlling for the patients’ baseline health status and characteristics of healthcare institutions in a series of multivariate regression models. This phenomenon is expected considering the findings from previous literatures [3,23,24].

Interestingly, while the likelihood of being hospitalized and the per capita utilization of inpatient services in terms of inpatient days and costs were significantly higher among MA program hypertension patients, the unit intensity of inpatient care (i.e., average LOS and cost per admission) was very similar or even lower in MA patients. This implies that the discrepancy in the utilization of inpatient services between the two groups may be mainly attributable to more frequent hospital admissions among the MA group, but may not be attributable to higher intensity or use of healthcare resources during hospitalization. The similar LOS and cost per admission suggests that the disease severity treated during hospitalization may not be higher in the MA group compared to the HI group. Another possibility is that the same standard of care is provided for hypertension-associated conditions in the inpatient setting in Korea regardless of the health plan in which patients are enrolled, resulting in similar LOS and cost per admission among all patients.

A comparison of the descriptive statistics of the patient characteristics considered in the present study indicates that the overall health status of the MA patients was worse than that of the HI patients. The MA patients were older, had higher comorbidity scores, incurred more medical costs, and had more ambulatory care visits than the HI patients. The discrepancy between the two groups with regard to baseline health status suggests that rigorous multivariate regression methods are essential to examine the unbiased independent relationship between the insurance type and the outcome variables. Therefore, in the present study we determined the most appropriate type of multivariate regression model for each outcome variable. For example, we chose the zero-inflated negative binomial model for the outcome variable of LOS in order to account for both overdispersion and excess zeroes. In addition, a generalized linear model with a log-link function was employed for the cost variable because of the nature of the cost data having high skewness and heteroscedasticity. A series of test statistics (i.e., the Vuong test, likelihood ratio test, and others) confirmed that these models were the best choices for each outcome variable.

We also attempted to adjust for patients’ baseline characteristics, health conditions, and institutional characteristics in the regression models using patient-level information available in the insurance claims records as much as possible. As a consequence, we observed a substantial reduction in differences in inpatient service utilization between the HI and the MA patients in the multivariate regression models. For instance, a three-fold difference in the hypertension-associated hospitalization rate between the two groups (62.29 per 1,000 patients for the HI group and 170.71 for the MA group, Table 2) was reduced to a 1.41- to 1.71-folddifference (presented as odds ratio, Table 3) after controlling the baseline heterogeneity between the two groups. For the hypertension-associated LOS, a difference of 4.76 times (1.27 days per patient for the HI group and 6.04 days for the MA group, Table 2) was reduced to 1.31 to 1.42 times (Table 3). Lastly, the hospitalization cost for hypertension-associated conditions was decreased to 1.093 to 1.412 times (Table 3) from 2.88 times (175,892 KRW per patient for the HI group and 507,192 KRW for the MA group, Table 2).

These observations suggest that differences in the baseline condition may play a substantial role in explaining why MA patients with hypertension tended to use more inpatient services than their counterparts in the HI program. However, although the extent of higher utilization of inpatient care by the MA group was decreased after adjusting for the differences in baseline health conditions, the positive associations between MA insurance status and higher utilization of inpatient services remained statistically significant for all three outcome variables. Therefore, based on the results of our empirical analysis, we can conclude that the initial hypothesis was supported: higher admission rates by MA patients persisted after adjustment for differences in baseline health condition and characteristics between the two groups, suggesting the overutilization of inpatient care in the treatment of hypertension for patients in the MA program.

It is possible that MA patients have higher rates of hospitalization because their primary care for hypertension is not sufficient or appropriate compared to HI patients. However, the utilization of ambulatory care services for cases with a primary diagnosis of hypertension was similar between the two groups: 4.3 outpatient visits for HI patients and 4.6 visits for MA patients during the baseline six-month period in this study. This does not support the hypothesis that insufficient primary care characterizes the treatment of hypertension in MA patients. There is no restriction in the type of healthcare institution that MA patients are allowed to use, meaning that both HI and MA patients have access to all medical institutions without restrictions. In addition, because the same list of insurance-covered services is available to treat both HI and MA patients under the NHI system in Korea, the possibility of discriminating behavior from physicians in choosing appropriate anti-hypertensive drugs depending on the insurance type (MA or HI) is considered very low. Unlike other conditions that require the use of expensive uninsured medicines and procedures, most antihypertensive medicines are covered by the NHI. Thus, inaccessibility to expensive non-covered medication cannot be a contributing factor in the different quality of care between MA and HI patients. However, although the same extent and quality of care may be provided, the patients’ poor self-management in terms of medication compliance, diet, and exercise may affect the quality of care and hospitalization. Consequently, we cannot confirm whether the higher utilization of hypertension-related inpatient services by MA patients is attributed to their unnecessary overutilization of the services or to the unsuccessful prevention of hospital admission due to the low quality of outpatient care for MA patients. Thus, we suggest a future study to identify the causes of higher utilization of inpatient services by hypertension patients in the MA program.

As the national health insurance system was employed in 1989 achieving universal healthcare coverage in Korea, we are facing fiscal challenges and various methods are being discussed [25,26]. Raising copayment rates or deductibles have been used as a means of reducing overutilization [23]. The evidence suggests that such plans would reduce healthcare utilizations [3,24]. However, this could be associated with worse outcomes if patients defer necessary care. Some evidence suggests that lowering copayments or deductibles would have positive health consequences or vice versa [27,28]. Thus, a careful approach should be employed to prevent overutilization of inpatient services after identifying the causes of overutilization in the MA program.

Several limitations of the present study should be addressed in future studies. First, the beneficiaries who switched between the HI and the MA program during the study period were not included in this study. This may affect the generalizability of the study findings. However, because the proportion of the MA enrollees in Korea has remained stable at around 3.5% for a decade, we believe that this limitation had little effect on the study findings [5]. Second, our study was restricted to patients who had claim records during both the six-month baseline period and the one-year follow-up period in order to provide complete information for the study subjects. This sampling process may have resulted in the inclusion of either sicker patients or more health-conscious patients. Third, the reliance on diagnostic codes to identify hypertension patients may have caused misclassification of the target patients due to the nature of claims data, such as voluntary or involuntary miscoding behavior. However, a recent validation study for the diagnostic codes of the NHI claims database in Korea addressed this concern. The validation study found that about 70% of the primary, secondary, or tertiary diagnosis codes from the NHI claims records coincided with those from medical records [29,30]. Fourth, due to the limited clinical information available in the claims database, we were not able to adjust the baseline severity of hypertension for each patient using laboratory information such as blood pressure. Instead, the Elixhauser comorbidity index and overall healthcare utilization observed during the baseline period were used as proxy measures to incorporate the overall health status of the individual patients into the analysis. Lastly, the ECM cost model may yield inconsistent estimates if the link function and underlying family distribution are not correctly specified and the cost estimation might be numerically unstable if there are many zeros [31]. However, we chose the link function and distribution based on statistical tests and these results might attenuate the concern over inconsistent estimates. In order to determine the appropriate relationship between the types of insurance and hospitalization costs, the patients with zero costs need to be included in the model. Because we found heteroscedasticity, using a model that requires transforming and retransforming the cost variable would have led to biased estimates. Thus, the cost model we used, which accommodated for skewness, was considered to be the most appropriate for examining the relationship between the insurance type and hospitalization costs.

## Conclusions

In conclusion, hypertension patients in the MA group showed a substantially higher utilization and cost of inpatient services for treating hypertension or associated conditions than those in the HI group. The finding of higher total hospitalization costs and LOS per patient, but similar unit cost and LOS per admission among MA patients, suggests that more frequent admissions are a major factor in the higher utilization of inpatient care in this group. Thus, focusing on methods to prevent episodes of hospital admission seems to be the most appropriate strategy for reducing the potential overutilization of inpatient care or low quality of primary care among hypertension patients in the MA program.
